# Pharmacokinetic Study of Epinephrine Hydrofluoroalkane (Primatene MIST) Metered-Dose Inhaler

**DOI:** 10.1089/jamp.2019.1577

**Published:** 2020-09-30

**Authors:** Edward M. Kerwin, Tony Marrs, Mary Z. Luo, Jack Y. Zhang

**Affiliations:** ^1^ Clinical Research Institute of Southern Oregon, Medford, Oregon, USA.; ^2^ Amphastar Pharmaceuticals, Inc., Rancho Cucamonga, California, USA.

**Keywords:** asthma, high dose, inhaled epinephrine, pharmacokinetic, systemic exposure

## Abstract

***Background*:** Primatene^®^ MIST CFC, an epinephrine metered-dose inhaler (MDI), was discontinued from the market owing to environmental concerns from its use of chlorofluorocarbon (CFC) propellant. As a result, a new epinephrine MDI was developed using hydrofluoroalkane (HFA) propellant. This article reports the pharmacokinetic (PK) profile of the newly Food and Drug Administration-approved epinephrine HFA MDI.

***Methods*:** A randomized, evaluator-blinded, active-controlled, single-dose, two-arm crossover study was conducted to evaluate the PK profile of epinephrine HFA (Primatene^®^ MIST) and epinephrine CFC (Primatene^®^ MIST CFC) in 23 healthy volunteers to characterize the epinephrine absorption extent and rate. The study was performed at a high dose of five times the normal dose to obtain measurable plasma epinephrine levels. Plasma epinephrine levels were measured and safety was assessed by adverse events (AEs), vital signs, clinical laboratory tests, and physical examinations.

***Results***: Epinephrine HFA demonstrated a greater systemic drug exposure (greater area under the curve) than that of epinephrine CFC (∼37% higher). The *C*_max_ occurred at ∼2 minutes and was significantly higher in the epinephrine HFA group (0.18 ng/mL) compared with the CFC version (0.046 ng/mL) at normal dose. Within 20 minutes, both groups demonstrated comparable plasma epinephrine levels. No clinically significant adverse effects were found to be associated with epinephrine HFA, even after an ultrahigh dose (i.e., 10 inhalations).

***Conclusions:*** The systemic exposure of epinephrine HFA was found to be higher for the first 20 minutes, and then comparable with epinephrine CFC. Minimal AEs were found in this study despite the very high 1250–2200 μg inhaled doses (i.e., 10 inhalations) used for PK characterization.

## Introduction

For several years, Primatene^®^ MIST CFC was the only over-the-counter (OTC) asthma epinephrine metered-dose inhaler (MDI) in the United States. Having been approved by the Food and Drug Administration in 1956, this product served a vital and unique role in meeting the OTC needs of asthma patients. However, Primatene^®^ MIST CFC was phased out in December 2011 by the Montreal Protocol because of environmental concerns from its chlorofluorocarbon (CFC) propellant.^(1)^ In response, a new epinephrine MDI (Primatene^®^ MIST) was developed that uses hydrofluoroalkane (HFA) propellant in place of CFC.

In addition to using different propellant, several other changes were made to the new formulation of Primatene^®^ MIST. Unlike the previous CFC version, which was a solution, Primatene^®^ MIST was formulated as a suspension. The new formulation provides enhanced drug delivery efficiency,^(2)^ allowing therapeutic efficacy to be achieved at a lower dose. The dose for Primatene^®^ MIST was reduced by 43%, from 220 μg/inhalation in the previous CFC version, to 125 μg/inhalation, with comparable efficacy.

At present, very few literatures were found clearly describing the pharmacokinetic (PK) profile of epinephrine MDIs. Because concerns exist surrounding the use of epinephrine as an asthma medication,^(3)^ the PK profile of the new epinephrine HFA MDI was explored in a PK clinical trial.

This article reports PK systemic exposure levels of high inhaled doses of the newly formulated Primatene^®^ MIST (epinephrine HFA, 10 × 125 μg) and adverse events (AEs) reported during the trial. The results are compared with an active control product, Primatene^®^ MIST CFC (10 × 220 μg).

## Materials and Methods

### Study design

This randomized, evaluator-blinded, active-controlled, single dose, two-arm crossover PK study in healthy subjects was conducted at a single site located in the United States. Because doses administered through inhalation may be too small for detection in plasma concentration,^(4)^ a high dose of five times the normal dose (i.e., 10 inhalations) was used to ensure adequate measurements of plasma concentrations for PK analysis. To thoroughly explore the PK profile of Epi-HFA, a stable isotope deuterium-labeled epinephrine was used to differentiate the administered exogenous epinephrine (epinephrine-d3) from the endogenous epinephrine (epinephrine-h3).

The study consisted of a screening visit and two study treatment visits separated by an intervisit interval of 3–14 days. During each study treatment visit, subjects self-administered 10 inhalations within 5 minutes of one of the randomized treatments: epinephrine HFA MDI (Epi-HFA, 125 μg/inhalation, total dose 1250 μg) and epinephrine CFC MDI (Epi-CFC, 220 μg/inhalation, total dose 2200 μg). Subjects were trained at screening and each study visit for correct dosing.

The study was conducted in accordance with Good Clinical Practice, including the International Conference on Harmonization Guidelines and the Declaration of Helsinki of the World Medical Association. The study protocol and informed consent form was approved by an institutional review board, and all subjects provided written informed consent before screening. The study was registered on the ClinicalTrials.gov (identifier NCT01188577).

### Study population

To allow a conservative evaluation, a sample size of 18 subjects was required. The study population comprised healthy men and nonpregnant women 18–30 years of age with a body mass index of 18.5–30.0 kg/m^2^, body weight ≥45 kg for women, and ≥50 kg for men, and sitting blood pressure ≤135/90 mmHg. Subjects demonstrated negative alcohol/drug screen tests, HIV, HBsAg, and HCV-Ab screen tests.

Subjects were ineligible if they had a smoking history of ≥10 pack-years, or lower respiratory tract infection within 4 weeks before screening. Subjects also were excluded if they had respiratory conditions, clinically significant cardiovascular and other systemic or organic illnesses which, per investigator discretion, may impact the subjects and/or the study. Other exclusions included use of prohibited medications, or intolerance to any of the study ingredients. Subjects could be discontinued from the study early at the discretion of the investigator owing to medical safety, noncompliance, or administrative concerns.

### PK assessments

Blood samples were collected at predose baseline (0 minutes) and at 2, 5, 7.5, 10, 12.5, 15, 20, 25, 30, and 45 minutes, and at 1, 1.5, 2, 4, and 6 hours postdose to construct the plasma concentration–time curve for PK analysis of epinephrine HFA-d3 (1250 μg) and epinephrine CFC (2200 μg), which served as active control.

At each PK sampling point, blood samples (∼10 mL) were collected in ice-chilled potassium–ethylenediaminetetraacetic acid sample tubes, each containing preadded 1% (V:V) 1.0 M sodium metabisulfite solution as an antioxidant. The sample tubes were well mixed immediately, kept on ice or refrigerated, and centrifuged within 60 minutes of collection. The harvested plasma from each sample were aliquoted to two storage tubes (∼2–2.5 mL/tube), and stored frozen at less than or equal to −20°C until analysis. These samples were tested by a validated liquid chromatography/mass spectrometry/mass spectrometry method with a calibration range of 20–2500 pg/mL and quantitative limit of 20 pg/mL (ppt). Plasma concentrations of epinephrine-d3 (exogenous) and epinephrine-h3 (endogenous) were analyzed for Epi-HFA.

For each PK concentration–time curve, including real (exogenous epinephrine) and apparent (total epinephrine) PK curves, the following parameters were obtained: the area under the plasma drug concentration–time curve (AUC) from time 0 (predose) until the last measurable drug concentration (area under the curve, AUC_0–*t*_), peak concentration from the PK curve (*C*_max_), and time corresponding to *C*_max_ (*t*_max_).

The primary PK endpoint was to compare the systemic exposure of Epi-HFA and Epi-CFC based on AUC_0–*t*_ of the apparent (total epinephrine) PK curves of Epi-HFA and that of Epi-CFC. The secondary endpoints included *C*_max_, *t*_max_, and relative bioavailability (RBA) between Epi-HFA versus Epi-CFC.

The plasma epinephrine data at normal doses was calculated based on the following formula:

$$C_{Total}^{Normal}\left(t\right)=\frac{C_{Total}^{High}\left(t\right)-b}{5}+b$$

where 1/5 denotes the fivefold ratio between high dose (10 puffs) and normal dose (2 puffs) and *b* denotes the endogenous epinephrine levels (based on average endogenous levels during the first hour after dosing).

### Safety measurements

Safety assessments included monitoring of AEs, vital signs including systolic blood pressure (SBP), diastolic blood pressure (DBP), and heart rate (HR), 12-lead electrocardiogram (ECG) (routine and QT/QTc intervals), clinical laboratory testing, and physical examinations were evaluated. AEs were coded using the Medical Dictionary for Regulatory Activities terminology.

### Statistical analysis

The primary analysis was to evaluate PK profile of Epi-HFA that includes the concentration–time curve and PK parameters such as AUC and *C*_max_. The main parameters were analyzed in all randomized subjects who received at least one dose of study drug treatment and had sufficient PK measurements. Pairwise comparisons of AUC_0–*t*_ between treatments were performed using one-sided *t*-tests with α = 0.05. Epi-HFA is comparable or has a significantly less systemic drug exposure than that for Epi-CFC if *p* ≤ 0.05. Similar statistical analysis was performed for other PK parameters including *C*_max_. Both real and apparent PK curves were fitted with the most suitable PK model by the standard PK analysis software Phoenix^®^ WinNonlin (Certara USA, Inc., Princeton, NJ).

Safety parameters were assessed in all randomized subjects who received at least one dose of study drug treatment.

## Results

### Subject demographics

A total of 35 subjects were screened and 23 (65.7%) were randomized to receive study treatment (Table 1). All the randomized subjects (100%) completed the study and were included in the PK and safety analyses. The majority of subjects were African American (60.9%) and men (73.9%) with the mean age of 24 years (Table 2).

**Table 1.** Subjects Disposition

No. of subjects screened	35	
No. of subjects randomized	23	
	*Epinephrine HFA-d3 1.25 mg*	*Epinephrine CFC 2.20 mg*
No. of subjects received study treatment(s)	23	23
No. of subjects completed study period with PK data available	23	23
No. of subjects completed study for crossover analysis vs. control	23	23

PK, pharmacokinetic; HFA, hydrofluoroalkane; CFC, chlorofluorocarbon.

**Table 2.** Demographic Data

Subject characteristics	Crossover (*n* = 23)
Age (years)	
Mean ± SD	23.8 ± 3.1
Range	18–29
Gender, %	
Male	73.9
Female	26.1
Weight (kg)	
Mean ± SD	72.0 ± 11.8
Range	54–92
Height (cm)	
Mean ± SD	172.5 ± 10.6
Range	153–194
Race group, %	
Caucasian	30.4
African American	60.9
Asian	8.7
Others	0.0

SD, standard deviation.

### PKs assessment

**FIG. 1.**
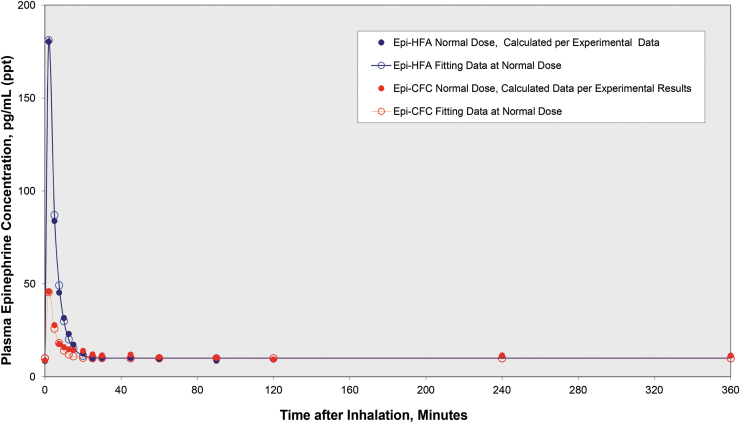
Extrapolated plasma epinephrine concentration curves at normal dose: Peak plasma epinephrine concentration levels occurred at 2 minutes postdose, 180 pg/mL for Epi-HFA and 46 pg/mL for Epi-CFC, respectively. HFA, hydrofluoroalkane; CFC, chlorofluorocarbon.

The plasma epinephrine levels for both exogenous epinephrine (epinephrine hfa-d3) and endogenous epinephrine for Epi-HFA at high dose are given in Table 3. For the active control, Epi-CFC, only total epinephrine (both exogenous and endogenous) was tested. Based on the high-dose experimental data, the plasma epinephrine levels at normal dose for Epi-HFA and Epi-CFC were extrapolated and the results are given in Table 3 and Figure 1.

**Table 3.** Plasma Epinephrine Concentration After High-Dose Inhalation and Normalized Dose

Time after inhalation (minutes)	Experimental data at high dose (ng/mL)				Calculated data at normal dose (ng/mL)	
	Exogenous Epi-HFA-d3	Endogenous Epi-HFA-h3	Total Epi-HFA 10 × 125 μg	Total Epi-CFC 10 × 220 μg	Epi-HFA 2 × 125 μg	Epi-CFC 2 × 220 μg
0	0	3	2.6	4.3	9	9
2	856	6	862	189	180	46
5	378	2	379	99	84	28
7.5	184	3	187	49	45	18
10	111	7	118	40	32	16
12.5	71	4	76	34	23	15
15	39	8	47	32	17	14
20	20	3	23	30	13	14
25	8	3	11	20	10	12
30	6	5	11	18	10	12
45	2	8	11	20	10	12
60	1	8	9	13	10	11
90	0	4	4	11	9	10
120	0	7	7	7	9	9
240	0	16	16	18	11	12
360	0	17	17	17	11	11

For total epinephrine, Epi-HFA demonstrated a greater systemic drug exposure (AUC) than that of Epi-CFC. The AUC for total epinephrine evaluated by statistical analysis was 8.5 ± 5.2 for Epi-HFA and 6.2 ± 4.1 ng/(mL·min) for Epi-CFC. Thus, the RBA of Epi-HFA was calculated to be 137%, which indicates that the AUC of Epi-HFA (1250 μg inhaled) was 37% greater than that of Epi-CFC (2200 μg inhaled) with a *p*-value of 0.052. Furthermore, the peak concentration (*C*_max_) in the blood system occurred at 2 minutes for Epi-HFA and was statistically greater (4.5 times) than Epi-CFC, with *p* < 0.0001 (Table 3). During this time, the plasma epinephrine levels that would result after a “normal” maximal dose of two puffs were calculated to be 180 pg/mL for Epi-HFA and 46 pg/mL for Epi-CFC. By 10 minutes after the *t*_max_ (at ∼12.5 minutes postdose), the plasma epinephrine level was reduced to about one-tenth of the *C*_max_ showing an elimination half-life (*t*_1/2_) of 2.6 minutes. The duration of higher plasma levels seen with Epi-HFA was brief (5–10 minutes), and plasma levels were found to be comparable with Epi-CFC by 20 minutes postdose and for all subsequent time points out to 360 minutes (Table 3). Within 60 minutes after inhalation, the radiolabeled exogenous epinephrine concentrations in plasma declined to an undetectable level in the blood plasma. The extrapolated PK curves for normal (two puff) doses of both Epi-HFA and Epi-CFC well matched a one-compartment model with the first-order input and first-order output (Fig. 1).

### Safety assessment

The overall extent of the study drug exposure for each subject was 1.25 mg of Epi-HFA and 2.20 mg of Epi-CFC delivered as single 10-puff doses on two separate treatment days. After completion of the crossover study visits, the total exposure of epinephrine inhaler was 3.45 mg.

A total of three AEs were reported, of which one (diarrhoea) was for Epi-HFA and two (cough and viral upper respiratory tract infection) for Epi-CFC. All AEs were classified as mild and not serious by the investigators. All were resolved without residual effects. The clinical laboratory data were within the normal range.

No significant effect to vital signs and ECG variables were found comparing high doses of Epi-HFA and Epi-CFC. The changes in vital signs and ECG were comparable with no substantial differences observed between the two study drug treatments. Both formulations of epinephrine did produce characteristic cardiovascular changes, with moderate increases in SBP and DBP and HR at 30 minutes postdose. Epi-HFA had a slightly higher increase in SBP and DBP (Table 4), and by 60 minutes, a normalization in both SBP and DBP was observed. There were two single cases of premature ventricular contractions (PVCs) in the Epi-HFA group that occurred with no arrhythmias. The PVC events occurred at time point of 6 hours postdose and were considered not clinically significant.

**Table 4.** Vital Signs and Electrocardiogram for High Dose of Study Drug Treatments

Items	Time after drug inhaled (minutes)	Epi-HFA-d3 1.25 mg,* n* = 23				Epi-CFC 2.20 mg,* n* = 23			
		Mean of data	Mean of Δ	Upper 95% CI of Δ	Mean Δ%	Mean of data	Mean of Δ	Upper 95% CI of Δ	Mean Δ%
SBP, mmHg	Baseline	115	—	—	—	116	—	—	—
	30	125	10.1	13.5	9.2	122	6.6	9.6	6.1
	60	115	−0.5	3.1	0.1	113	−2.7	1.8	−1.6
	360	122	6.8	10.2	6.2	121	5.2	7.0	4.7
DBP, mmHg	Baseline	60	—	—	—	60	—	—	—
	30	64	4.5	7.5	8.3	63	2.6	5.0	4.4
	60	62	2.7	6.9	5.8	59	−1.4	1.0	−1.9
	360	61	1.8	4.4	4.2	62	2.1	3.7	3.8
HR, bpm (by vital sign)	Baseline	61	—	—	—	61	—	—	—
	30	65	4.3	6.0	7.0	65	4.2	6.7	7.4
	60	63	2.1	3.9	3.4	61	0.0	2.0	0.1
	360	65	3.9	5.7	6.7	66	5.6	6.9	9.4
HR, bpm (by ECG)	Baseline	60	—	—	—	61	—	—	—
	30	64	3.3	5.1	5.6	64	3.2	5.5	6.1
	360	67	6.6	9.1	11.4	65	4.7	6.7	7.8
QT, ms	Baseline	406	—	—	—	404	—	—	—
	30	397	−9.5	−1.4	−2.2	395	−8.9	−4.1	−2.1
	360	387	−19.0	−10.0	−4.5	391	−13.0	−6.7	−3.3
QTc, ms	Baseline	402	—	—	—	401	—	—	—
	30	403	1.4	5.8	0.4	404	2.3	7.9	0.6
	360	403	1.7	6.3	0.5	402	0.7	5.5	0.2
QTc-B, ms	Baseline	403	—	—	—	402	—	—	—
	30	404	1.2	5.6	0.3	405	2.4	8.0	0.6
	360	404	1.5	6.2	0.4	403	0.8	5.7	0.2
QTc-F, ms	Baseline	404	—	—	—	402	—	—	—
	30	401	−2.4	2.6	−0.5	401	−1.3	2.6	−0.3
	360	398	−5.5	−0.6	−1.3	398	−4.0	0.5	−1.0

SBP, systolic blood pressure; DBP, diastolic blood pressure; HR, heart rate; CI, confidence interval; ECG, electrocardiogram.

## Discussion

With respect to PKs, the overall systemic exposure of epinephrine HFA was found to be low. The *C*_max_ in both Epi-HFA and Epi-CFC occurred at 2 minutes (*t*_max_) postdose and was higher in Epi-HFA (180 pg/mL) at normal dose compared with that for Epi-CFC (46 pg/mL). Although plasma epinephrine levels were relatively higher in the Epi-HFA group, this was found to be a transient effect occurring in the first few minutes after drug administration. Systemic epinephrine levels decreased rapidly with a half-life of 2.6 minutes and were comparable between the two study drugs by 20 minutes postdose. Despite the higher *C*_max_, minimal adverse effects were observed in Epi-HFA even after administration of very high doses (10 inhalations).

One possible explanation for the low occurrence of adverse effects is that when considering that the typical blood volume for an adult is 4.7–5 L, the amount of epinephrine distributed into the adult blood system from Epi-HFA at peak *C*_max_ (180 pg/mL) would only be 0.36% (0.9 μg/250 μg) of the total amount of epinephrine inhaled. This suggests that most of the epinephrine may be retained and locally metabolized in the patients' lungs, or it may be absorbed over several hours. Furthermore, this amount (180 pg/mL) does not appear to be a safety concern, as it represents only ∼20% of the normal human endogenous epinephrine levels measurable during vigorous exercise,^*^ 5 nmol/L or 0.92 ng/mL.^(5,6)^

The bioavailability and cardiovascular results found in this study were consistent with past publications on the inhalation of epinephrine, which did not find significant safety risks with epinephrine MDI even at high dosages.^(7,8)^ In the previous study of epinephrine HFA at high dose (i.e., 10 inhalations), an increase in systolic and diastolic blood pressure and HR were noted at 10 minutes after dosing that returned to baseline by 360 minutes.^(9)^ Similar to the effects seen in this study, these changes in vital signs are associated with the use of epinephrine HFA beyond the labeled dose, which was taking 10 inhalations of 125 μg in rapid succession. The changes in blood pressure and HR are expected to be minimal with the labeled dose.

Despite the common concern regarding the cardiovascular effects of epinephrine,^(3)^ data on the serious side effects of epinephrine metered-dose inhalation in the literature are scarce. Serious effects were observed when epinephrine was given by injection where systemic bioavailability of the drug approaches 100%.^(10,11)^ A safety review on epinephrine found that the most serious cardiac adverse effects occur when epinephrine is given by intravenous injection (e.g., severe myocardial ischemia).^(11)^ Therefore, a future study exploring the PK safety between epinephrine MDIs and epinephrine injection will be worthwhile.

A potential limitation of this study is that it was conducted in healthy subjects instead of asthmatic patients in whom the drug will be used. Furthermore, the study design was restricted to subjects at the ages of 18–30. Therefore, further studies evaluating subjects at a wider age range would provide a broader PK analysis.

In conclusion, the systemic exposure of epinephrine HFA was found to be low in this PK study, with levels similar to epinephrine CFC after the initial 10–20 minutes postdose. There was an increased cardio-dynamic effect noted at 30 minutes postdose with both high-dose inhaled formulations, but few AEs and no clinically important safety findings were reported in this study.

## Notes

^*^ Exercise level is defined as 110% max volume of oxygen (VO_2_max), that is, when running 2 minutes at the speed of 9.7 km/h for 323 m, the epinephrine level is in the range of 0.67–1.6 ng/mL.^6^

## Notes

Reviewed by:

Tunde Otulana

Gur Jai Pal Singh
